# Structural basis of chemokine recognition by the class A3 tick evasin EVA‐ACA1001

**DOI:** 10.1002/pro.4999

**Published:** 2024-05-09

**Authors:** Shankar Raj Devkota, Pramod Aryal, Matthew C. J. Wilce, Richard J. Payne, Martin J. Stone, Ram Prasad Bhusal

**Affiliations:** ^1^ Department of Biochemistry and Molecular Biology, Monash Biomedicine Discovery Institute Monash University Clayton VIC Australia; ^2^ School of Chemistry The University of Sydney Sydney NSW Australia; ^3^ Australian Research Council Centre of Excellence for Innovations in Peptide and Protein Science The University of Sydney Sydney NSW Australia

**Keywords:** inflammation, chemokines, tick evasins

## Abstract

Ticks produce chemokine‐binding proteins, known as evasins, in their saliva to subvert the host's immune response. Evasins bind to chemokines and thereby inhibit the activation of their cognate chemokine receptors, thus suppressing leukocyte recruitment and inflammation. We recently described subclass A3 evasins, which, like other class A evasins, exclusively target CC chemokines but appear to use a different binding site architecture to control target selectivity among CC chemokines. We now describe the structural basis of chemokine recognition by the class A3 evasin EVA‐ACA1001. EVA‐ACA1001 binds to almost all human CC chemokines and inhibits receptor activation. Truncation mutants of EVA‐ACA1001 showed that, unlike class A1 evasins, both the N‐ and C‐termini of EVA‐ACA1001 play minimal roles in chemokine binding. To understand the structural basis of its broad chemokine recognition, we determined the crystal structure of EVA‐ACA1001 in complex with the human chemokine CCL16. EVA‐ACA1001 forms backbone‐backbone interactions with the CC motif of CCL16, a conserved feature of all class A evasin‐chemokine complexes. A hydrophobic pocket in EVA‐ACA1001, formed by several aromatic side chains and the unique disulfide bond of class A3 evasins, accommodates the residue immediately following the CC motif (the “CC + 1 residue”) of CCL16. This interaction is shared with EVA‐AAM1001, the only other class A3 evasins characterized to date, suggesting it may represent a common mechanism that accounts for the broad recognition of CC chemokines by class A3 evasins.

## 1 INTRODUCTION

Inflammation is characterized by the accumulation of leukocytes in damaged or injured tissues. Chemokines mediate leukocyte recruitment by interacting with chemokine receptors located on leukocyte cell membranes (Griffith et al., 2014; Stone et al., 2017). Chemokines and their receptors play essential roles in development and homeostasis, while their dysregulation results in the progression of various autoimmune and inflammatory diseases (Chen et al., 2018). Blocking selected chemokine‐chemokine receptor interactions is a promising anti‐inflammatory therapeutic strategy but may require agents that selectively target multiple chemokines or receptors.

Various parasitic organisms, such as ticks, worms, and viruses, evade the inflammatory responses of their hosts by secreting immunomodulatory proteins, including chemokine‐binding proteins (Déruaz et al., 2008; Hewitson et al., 2009; Maizels & McSorley, 2016). In particular, evasins (designated by the prefix “EVA”) are produced in the saliva of ticks and bind to chemokines, thus preventing chemokine receptor activation and the downstream inflammatory cascade (Bhusal et al., 2020). EVA‐1, EVA‐3, and EVA‐4, from the tick *Rhipicephalus sanguineus* have exhibited potent anti‐inflammatory activity in various murine inflammation models (Bonvin et al., 2016; Déruaz et al., 2013; Montecucco et al., 2010; Montecucco et al., 2014; Russo et al., 2011). Bioinformatics searches have identified several hundred evasin‐like molecules, of which a few dozen have now been validated as chemokine‐binding proteins (Hayward et al., 2017; Lee et al., 2019; Singh et al., 2017).

Evasins are classified into class A or class B based on their sequence similarity and the chemokines to which they bind (Bhusal et al., 2020). Evasins belonging to class A bind exclusively to CC chemokines, whereas those belonging to class B bind exclusively to CXC chemokines. Recently, class A has been subclassified into classes A1, A2, and A3 according to the number of conserved cysteines (Devkota et al., 2023). Class A1 evasins such as EVA‐1, EVA‐4, and EVA‐P974 (Bhusal et al., 2022; Denisov et al., 2020; Dias et al., 2009) possess eight cysteines that form four conserved disulfide bonds, whereas class A2 evasins have six cysteines and lack one of the disulfide bonds (Bhattacharya & Nuttall, 2021). We recently described class A3 evasins, which have 10 cysteines and an additional disulfide, and reported chemokine‐bound structures of the class A3 evasin EVA‐AAM1001 (Devkota et al., 2023). We found that, unlike the recognition mechanism of class A1 evasins, the N‐ and C‐termini of EVA‐AAM1001 make minimal contribution to chemokine binding affinity; instead, EVA‐AAM1001 utilizes a distinct hydrophobic pocket to interact with the CC + 1 residue of target CC chemokines (Devkota et al., 2023). We now describe a structure–function analysis of EVA‐ACA1001, a class A3 evasin from *Amblyomma cajennense* (Cayenne tick). We find that EVA‐ACA1001 binds to multiple CC chemokines with high affinity and inhibits chemokine receptor activation. A structure–function study of EVA‐ACA1001 reveals a mode of chemokine binding that is consistent with EVA‐AAM1001 and thus suggests that there is a similar mode of binding for this class that may account for the broad chemokine recognition properties.

## 2 RESULTS

### 2.1 EVA‐ACA1001 is a broad chemokine binder

**FIGURE 1:**
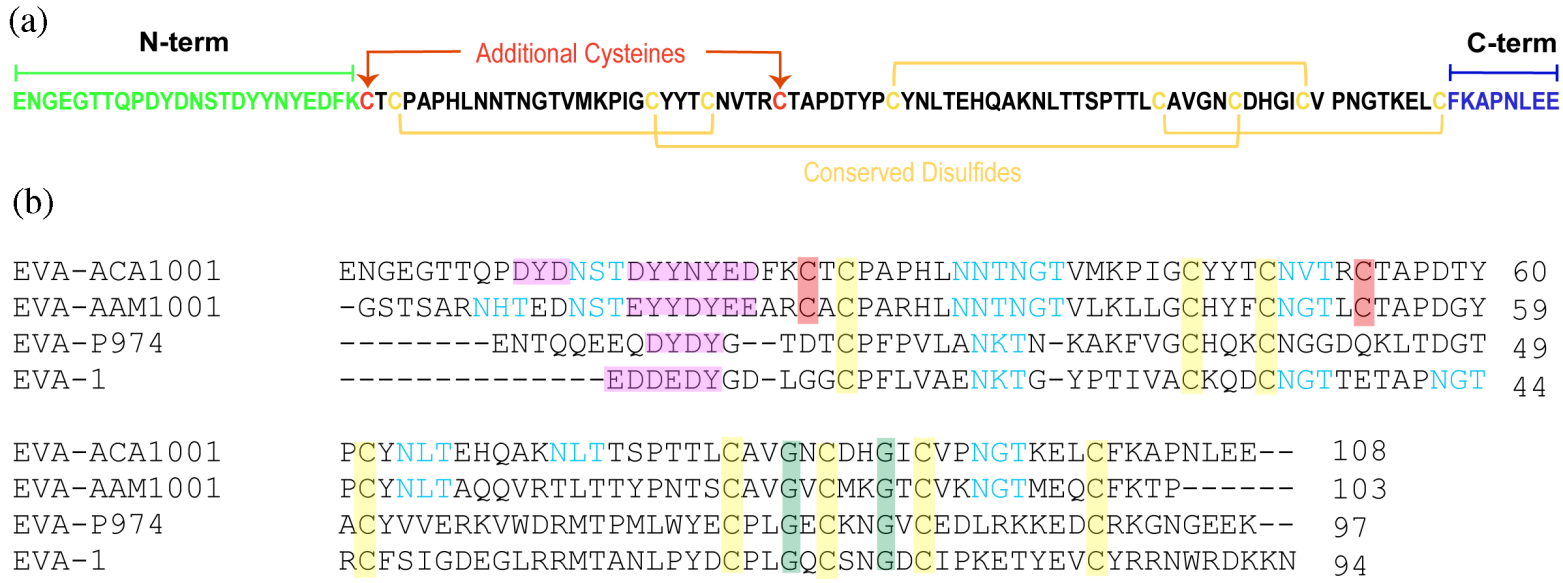
Sequence features of EVA‐ACA1001. (a) EVA‐ACA1001 sequence showing the conserved disulfide bonds in subclass A1 and A3 evasins (yellow), the additional disulfide pair unique to subclass A3 evasins (red), the N‐terminus (green), and the C‐terminus (blue). (b) Multiple sequence alignment (obtained using Clustal Omega) of the class A3 evasins EVA‐ACA1001 and EVA‐AAM1001 and the class A1 evasins EVA‐1 and EVA‐P974. Highlighted residues are conserved cysteines (yellow), additional cysteines in class A3 evasins (red), conserved glycines (green), putative tyrosine sulfation sites (magenta), and potential N‐linked glycosylation sites (cyan).

**FIGURE 2:**
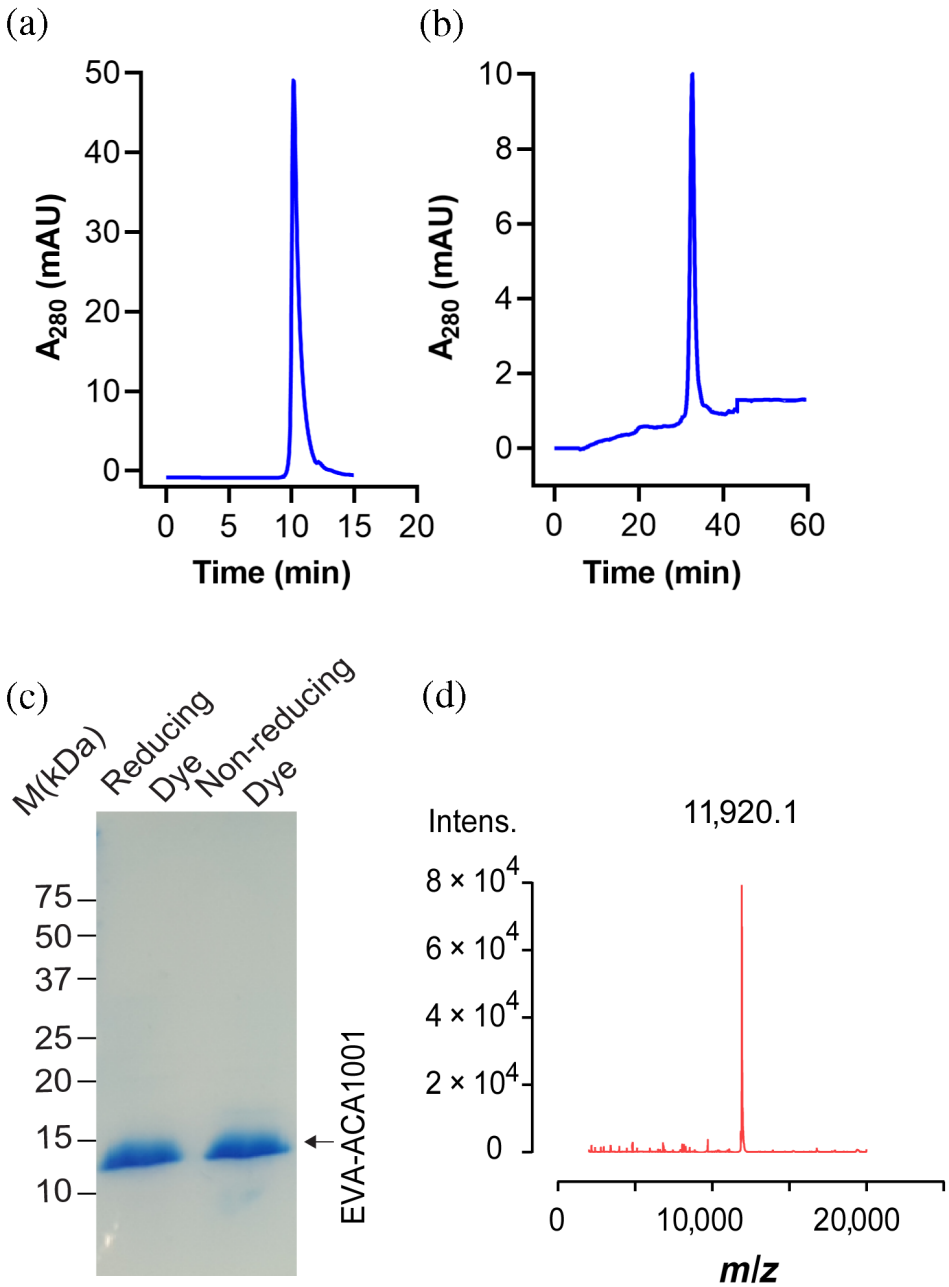
Quality control analysis of purified EVA‐ACA1001. (a) Analytical size‐exclusion chromatography (column: Yarra TM 3 μm SEC‐2000 Phenomenex; eluent: 0.1 M NaH_2_PO_4_ pH 7.5; and flow rate: 0.35 mL/min) (b) analytical reversed‐phase high‐performance liquid chromatography (column: Protein C4 214TP5315 GRACE VYDAC; eluent: a mobile phase of 0.1% trifluoroacetic acid in water (Solvent A) and 0.1% trifluoroacetic acid in acetonitrile (Solvent B) with a linear gradient of 0–60% B over 60 min; and flow rate: 0.35 μL/min). (c) Coomassie blue‐stained SDS‐polyacrylamide gel showing purified EVA‐ACA1001 in reducing and nonreducing conditions; protein molecular weight markers are shown as black lines and labeled in kDa; lane 1, EVA‐ACA1001 in reducing conditions; and lane 2, EVA‐ACA1001 in nonreducing conditions. (d) Analysis of EVA‐ACA1001 by mass spectrometry. Purified EVA‐ACA1001 was analyzed by liquid chromatography‐electrospray ionization mass spectrometry (LC MS‐ESI). The expected mass of EVA‐ACA1001 with five disulfide bonds is 11,921.14 Da.

EVA‐ACA1001 (UniProt accession code: A0A023FFD0, previously known as Evasin P991) (Singh et al., 2017) possesses the pattern of 10 cysteine residues that we recently identified as being characteristic of class A3 evasins (Figure 1a), along with other conserved features of all class A evasins, including conserved glycine residues and likely tyrosine sulfation and N‐linked glycosylation sites (Figure 1b). To enable structural characterization, EVA‐ACA1001 was overexpressed in *Escherichia coli* and purified using chromatographic techniques. The purified EVA‐ACA1001 showed a single sharp peak in analytical size exclusion chromatography and analytical HPLC (Figure 2a,b) and a single band by SDS‐PAGE (Figure 2c). Moreover, LC–MS revealed a molecular mass of 11,920 ± 1 Da (Figure 2d), consistent with the presence of the five predicted disulfide bonds (calculated mass 11,921 Da).

**FIGURE 3:**
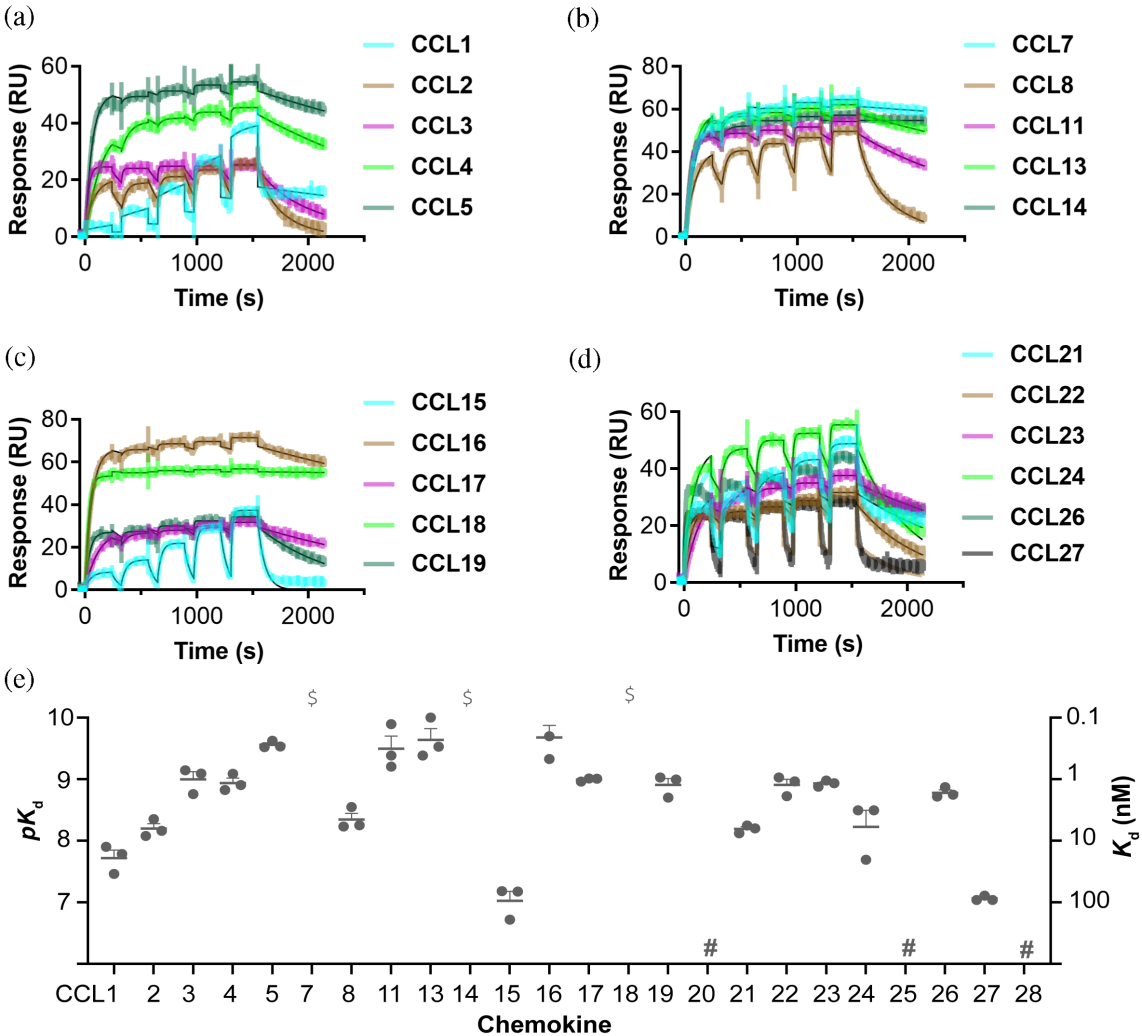
Purified EVA‐ACA1001 binds to CC chemokines. (a–d) Representative surface plasmon resonance (SPR) sensorgrams show the binding of immobilized EVA‐ACA1001 to five increasing concentrations of chemokines (31.25, 62.5, 125, 250, and 500 nM). (e) Binding affinities (left axis: *pK*_d_ = −log[*K*_d_, *M*]; right axis: *K*_d_, nM) of EVA‐ACA1001 for CC chemokines, measured by SPR. Data are presented as mean *pK*_d_ ± SEM from three independent experiments. $, *K*_d_ <0.1 nM; #, no measurable binding at 500 nM chemokine concentration.

We used surface plasmon resonance (SPR) to screen the binding of EVA‐ACA1001 to all human chemokines at a single concentration of chemokines (500 nM). The data showed that EVA‐ACA1001 bound to 21 of 24 human CC chemokines, but none of the 15 CXC, one CX_3_C, or one XC chemokines tested (Figure S1). The binding affinity was determined for these 21 CC chemokines by SPR using multiple concentrations of chemokines (Figure 3a–d); the binding affinities (Figure 3e, Table 1) varied from ~100 nM (CCL15 and CCL27) to tighter than 0.1 nM (CCL7, CCL14, and CCL18).

**TABLE 1:** Binding affinities (*K*_d_ in nM) of EVA‐ACA1001 to human chemokines measured by surface plasmon resonance using multiple‐cycle kinetics.

Chemokine	*pK*_d_ ± SEM (*K*_d_ nM)
CCL1	7.71 ± 0.13 (21.1)
CCL2	8.19 ± 0.08 (6.5)
CCL3	8.99 ± 0.12(1.08)
CCL4	8.93 ± 0.07 (1.18)
CCL5	9.55 ± 0.03 (0.27)
CCL7	^a^
CCL8	8.34 ± 0.10 (4.76)
CCL11	9.49 ± 0.20 (0.38)
CCL13	9.41 ± 0.05 (0.39)
CCL14	^a^
CCL15	7.02 ± 0.15 (107)
CCL16	9.34 ± 0.19 (0.54)
CCL17	8.99 ± 0.01 (1.01)
CCL18	^a^
CCL19	8.90 ± 0.10 (1.31)
CCL20	^b^
CCL21	8.19 ± 0.03 (6.50)
CCL22	8.90 ± 0.09 (1.29)
CCL23	8.93 ± 0.02 (1.17)
CCL24	8.22 ± 0.26 (8.90)
CCL25	^b^
CCL26	8.77 ± 0.04 (1.68)
CCL27	7.07 ± 0.02 (84.8)
CCL28	^b^

*Note*: Data are reported as mean ± SEM (*n* = 3); the corresponding *K*_d_ values (in nM) are given in parentheses.

^a^ *K*_d_ < 0.1 nM.

^b^ No measurable binding at 500 nM chemokine concentration.

**FIGURE 4:**
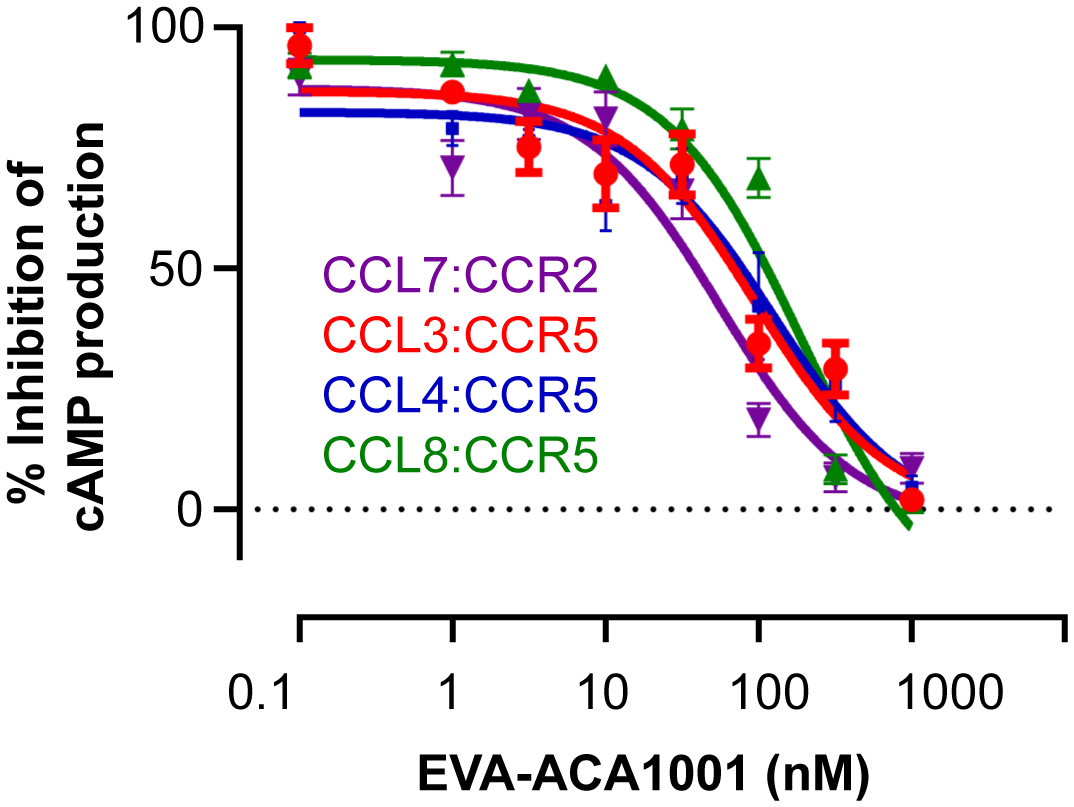
Purified EVA‐ACA1001 inhibits the function of chemokines. Concentration‐dependent inhibition of chemokine‐mediated activation of the indicated chemokine receptors by EVA‐ACA1001. Flp‐In‐CHO cells stably expressing CCR2 or CCR5 were transiently transfected with the CAMYEL biosensor and treated, sequentially, with the Rluc substrate coelenterazine‐h (5 μM), chemokines (100 nM CCL2; 60 nM CCL3; 80 nM CCL4; or 100 nM CCL8) in the presence of the indicated concentrations of EVA‐ACA1001, and forskolin to stimulate cyclic AMP (cAMP) synthesis. The resulting CAMYEL BRET ratio is expressed as the percentage inhibition of forskolin‐induced cAMP production by chemokines. The data presented are the mean ± SEM of at least three independent measurements.

We next evaluated the ability of EVA‐ACA1001 to inhibit the activity of selected chemokines in cell‐based receptor‐activation assays. Chemokine receptors activate their cognate receptors to induce G protein‐dependent and independent signaling pathways. We detected the activation of CCR2 and CCR5 by monitoring chemokine‐stimulated (G_αi_‐dependent) inhibition of cyclic AMP (cAMP) production in CHO cells that stably expressed the chemokine receptor and had been transiently transfected to express a cAMP biosensor. In this assay, chemokines (CCL3, CCL4, CCL7, and CCL8) inhibited the synthesis of cAMP by activating their cognate receptors. EVA‐ACA1001 mitigated this effect in a concentration‐dependent manner (Figure 4, Table 2). The CCL7‐stimulated activation of CCR2 was inhibited by EVA‐ACA1001 with a 50% inhibitory concentration (*IC*_*50*_) of ~55 nM, whereas the activation of CCR5 stimulated by CCL3, CCL4, or CCL8 was inhibited by EVA‐ACA1001 with *IC*_*50*_ values of ~94 nM, ~84 nM, or ~189 nM, respectively. These *IC*_*50*_ values correspond well to the chemokine concentrations used in these assays, as the *K*_d_ values for EVA‐ACA1001:chemokine binding are below the chemokine concentrations used. These findings show that EVA‐ACA1001 binds to CC chemokines, resulting in inhibition of their receptor‐mediated activity.

**TABLE 2:** Inhibition constants for inhibition of chemokine activity by EVA‐ACA1001.

Chemokine	*pIC*_*50*_ (M)	*IC*_*50*_ (nM)
CCL3	7.02 ± 0.12	94
CCL4	7.07 ± 0.17	84
CCL7	7.25 ± 0.11	55
CCL8	6.51 ± 0.11	188

*Note*: CCL3, CCL4, and CCL8 signaling at CCR2 and CCL7 signaling at CCR5 were measured as inhibitions of cyclic AMP synthesis. Inhibition constants are reported as *pIC*_*50*_ = −log(*IC*_*50*_, *M*) ± SEM (*n* = 3); the corresponding *IC*_*50*_ values (in nM) are given in parentheses.

### 2.2 The core of EVA‐ACA1001 confers broad chemokine binding

**FIGURE 5:**
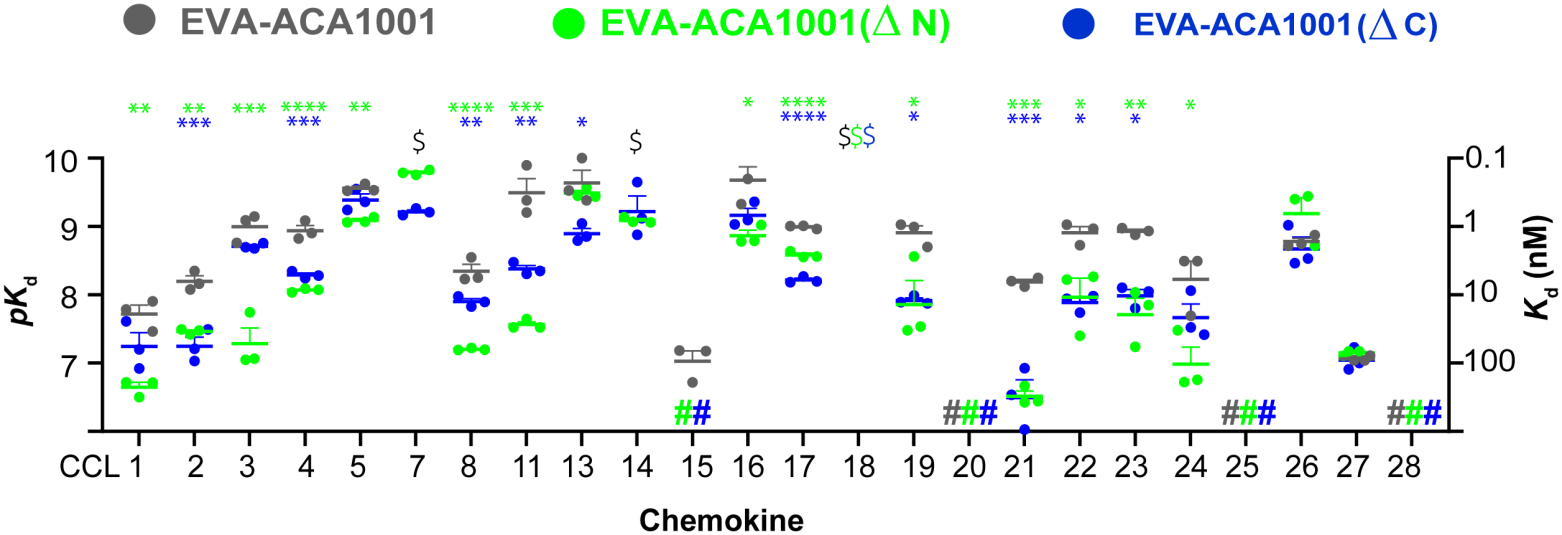
Truncation of EVA‐ACA1001 termini has minimal effects on binding to chemokines. N‐terminal (green) or C‐terminal (blue) truncation of EVA‐ACA1001 has only small effects on chemokine binding affinity or selectivity. Binding affinities (left axis: *pK*_d_ = −log[*K*_d_, *M*]; right axis: *K*_d_, nM) are presented as mean *pK*_d_ ± SEM from three independent experiments. $, *K*_d_ <0.1 nM; #, no measurable binding at 500 nM chemokine concentration; *p < 0.05, **p < 0.01, ***p < 0.001, ****p < 0.0001, compared to full‐length EVA‐ACA1001 (one‐way ANOVA with Šídák correction for multiple comparisons for each chemokine; or a two‐tailed *t*‐test if only a single comparison was possible).

For all the class A1 evasins studied in detail to date, the N‐ and/or C‐termini make substantial contributions to chemokine binding affinity (17–20). However, we found that the termini of the first‐class A3 evasin, EVA‐AAM1001, could be deleted with only minor effects on chemokine binding. Therefore, to determine the role of the N‐ and C‐ termini in the chemokine binding affinity and selectivity of EVA‐ACA1001, we made two variants by completely deleting the N‐ or C‐termini (Figure 1a) and determined their chemokine binding affinities using SPR (Figure 5). Complete deletion of the N‐terminal residues, giving EVA‐ACA1001(ΔN), had small (albeit significant for some chemokines) effects on chemokine binding affinity. Similarly, complete deletion of the C‐terminus, to give EVA‐ACA1001(ΔC), caused only small reductions in binding affinities. These results indicate that the majority of the chemokine binding energy of EVA‐ACA1001 is likely to result from the structure and interactions of amino acid residues 25–100, rather than the N‐ and C‐ terminal regions.

### 2.3 Structure of EVA‐ACA1001 in complex with CCL16

**FIGURE 6:**
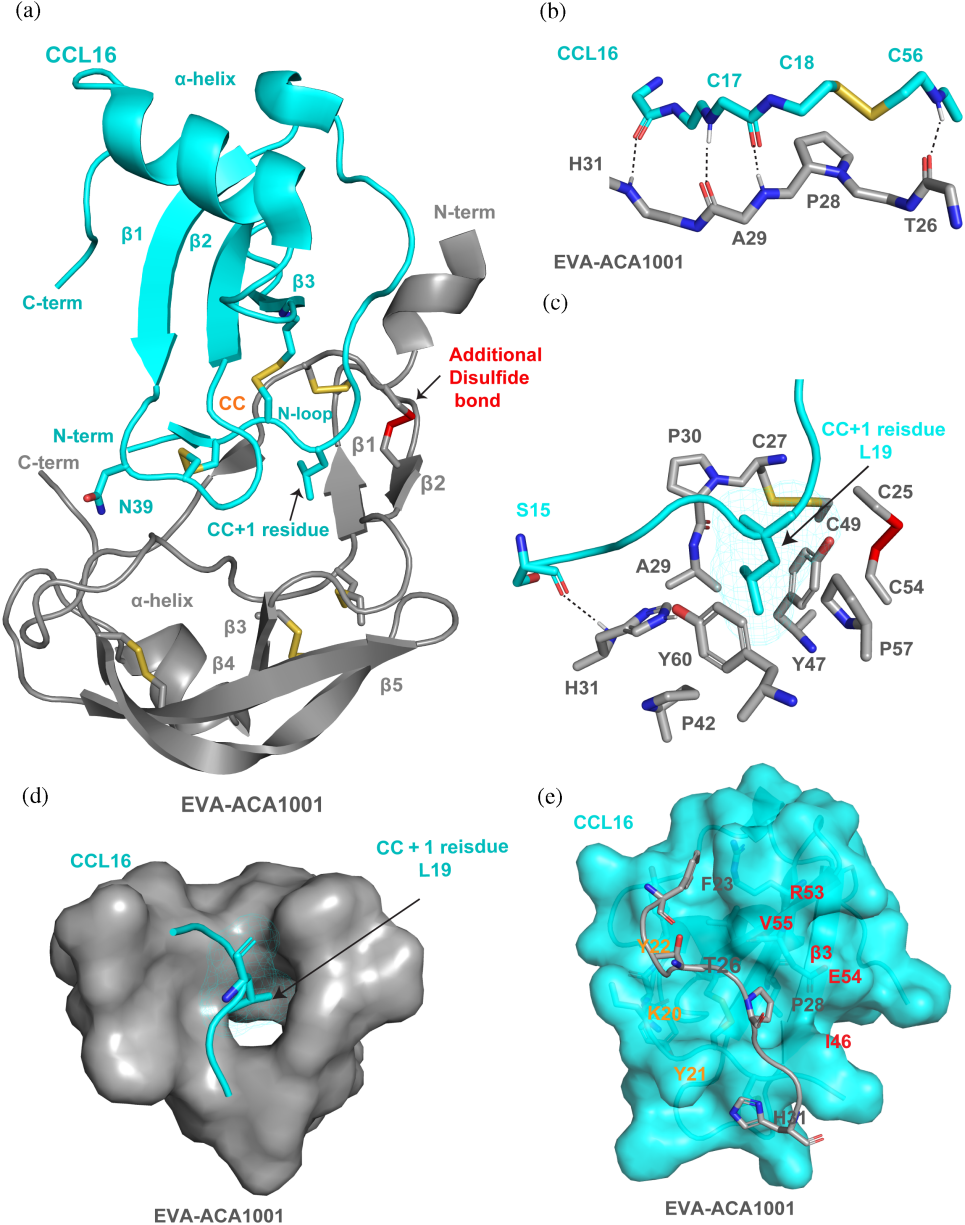
Structural basis of broad‐spectrum chemokine binding by EVA‐ACA1001. (a) Structure of EVA‐ACA1001 in complex with CCL16. EVA‐ACA1001 is shown as gray ribbons; disulfides conserved with subclass A1 evasins are shown as yellow sticks; the unique disulfide in subclass A3 evasins is shown as red sticks. CCL16 is shown as cyan ribbons; conserved disulfides are shown as yellow sticks. Selected residues (the CC + 1 residue, Leu19 and Asn39) are also shown as sticks. (b) Backbone‐backbone hydrogen bond interactions between the β1 region of EVA‐ACA1001 and the CC motif (and adjacent residues) of CCL16. (c) Interaction of the EVA‐ACA1001 hydrophobic pocket (colored gray, blue, red, and yellow, by atom type) with the CCL16 CC + 1 residue, Leu19 (cyan). (d) Surface representation showing the EVA‐ACA1001 binding pocket (gray surface) holding the CCL16 CC + 1 residue, Leu19 (sticks). (e) The EVA‐ACA1001 extended region at the junction between the N‐terminus and the core (gray) forms interactions with a shallow groove formed by the N‐loop (Lys20‐Tyr22) and the β3‐strand (Ile46‐Arg53) of CCL16, shown as a space‐filling model in cyan; important residues of the N‐loop (orange) and the β3‐strand (red) are labeled.

To understand how the core of EVA‐ACA1001 defines broad‐spectrum binding, we determined the crystal structure of EVA‐ACA1001 in complex with the human chemokine CCL16 to a resolution of 2.7 Å resolution (Table 3, Figure 6a). The complex crystallized in 1:1 stoichiometry with two molecules per asymmetric unit. In both complexes, the extreme N‐ and C‐termini of both EVA‐ACA1001 and CCL16 were not clearly visible in the electron density maps, consistent with the minimal contributions of these regions to binding. Additionally, we generated an AlphaFold model of EVA‐ACA1001 in complex with CCL16 and compared it with our experimental structure. The alignment revealed a close structural similarity between the two structures (Figure S2a). Furthermore, we generated AlphaFold models of EVA‐ACA1001 with four other CC chemokines (CCL1, CCL5, CCL7, and CCL15), all of which exhibited a similar fold and mode of interactions (Figure S2b–e). These findings collectively indicate that AlphaFold models can be valuable tools for gaining a comprehensive understanding of evasins' interactions with CC chemokines.

**TABLE 3:** X‐ray diffraction data collection and refinement statistics.

	EVA‐ACA1001:CCL16
PDB ID	8FK9
Data collection	
Beamline	Australian Synchrotron MX2
Wavelength (Å)	0.953
Resolution range (Å)	49.24–2.70 (2.83–2.70)
Space group	C 2 2 2_1_
Cell dimensions	
*a*, *b*, *c* (Å)	117.0, 122.8, 60.5
*α*, *β*, *γ* (^0^)	90, 90, 90
*R*_merge_	0.489 (3.946)
*R*_meas_	0.509 (4.089)
*R*_pim_	0.139 (1.068)
*I/s*(*I*)	7.0 (1.8)
CC_1/2_	0.987 (0.592)
Completeness (%)	99.9 (100)
Total reflections	160,050 (23,048)
Unique reflections	12,337 (1609)
Multiplicity	13.0 (14.3)
Refinement	
*R*_work_ */R*_free_	0.244/0.264
No. of atoms	2208
Macromolecules	2181
Solvent	27
Average *B* factors	54.7
Macromolecules	54.8
Solvent	49.8
r. m. s. deviations	
Bond lengths (Å)	0.003
Bond angles (^0^)	0.77
Ramachandran statistics (%)	
Favored	95.49
Allowed	4.17
Outliers	0.35
Rotamer outliers	0.00
Clash score	6.48
MolProbity score	1.67

*Note*: Values in parentheses are for the outermost resolution shell.

EVA‐ACA1001 comprises one β‐strand forming an anti‐parallel beta sheet with the CC motif of CCL16, two antiparallel β‐sheets, and two α‐ helices. As expected, the structure contains five intramolecular disulfide bonds, four (Cys27‐Cys49, Cys45‐Cys86, Cys62‐Cys91, and Cys81‐Cys100) of which are conserved in all class A evasins, and the fifth (Cys25‐Cys54) that is unique to class A3 evasins (Figure 6a). Residues Leu32 to Lys41 form a hairpin‐like structure (Figure S3) with a similar conformation to a beta‐hairpin in other evasin structures. However, this region does not meet the criteria for being assigned a beta sheet. Consequently, the strands labeled β3–β7 in EVA‐1, EVA‐P974, and EVA‐AAM1001 are labeled β1–β5, respectively, in EVA‐ACA1001. CCL16 adopts the canonical chemokine architecture, which consists of three β‐strands and an α‐helix linked via two conserved disulfide bonds to the disordered N‐terminus, the CC motif, and the irregularly‐structured “N‐loop” (Figure 6a) (Weiergräber et al., 2022). EVA‐ACA1001 interacts with the chemokine CC motif, the residue immediately following the CC motif (“CC + 1” residue), and a shallow groove between the N‐loop and the third β‐strand (β3). Considering that all these interacting chemokine regions are also involved in chemokine receptor binding, the structure is consistent with the ability of EVA‐ACA1001 to inhibit chemokine receptor activation.

The CC motif of CCL16 interacts with EVA‐ACA1001 through four backbone‐backbone hydrogen bonds (Figure 6b). These interactions are also observed in all other reported structures of class A1 and A3 evasins bound to chemokines and are thought to be critical for specific recognition of CC chemokines rather than other classes of chemokines (Bhusal et al., 2022). In addition to these conserved interactions, Asn39 of CCL16, adjacent to Cys38 (which is disulfide bonded to the CC motif), forms hydrogen bonds with both Thr38 and Met40 of EVA‐ACA1001 (Figure S4). The CC + 1 residue (Leu19) of CCL16 is located within a deep hydrophobic pocket in the core of EVA‐ACA1001 (Figure 6c, d). The hydrophobic pocket is defined by the side chains of Cys27, Ala29, Pro30, His31, Pro42, Tyr47, Cys49, Pro57, and Tyr60 and by the Cys25–Cys54 disulfide bond, the unique disulfide in class A3 evasins (Figure 6c, Figure S5). Notably, His31, Pro42, and Tyr60 form a hydrophobic cluster at the base of the hydrophobic pocket, which differs from the equivalent region of EVA‐AAM1001 and may contribute to the broader spectrum of chemokine binding by EVA‐ACA1001 (see Discussion). Indeed, the ability of this pocket to efficiently accommodate aromatic CC + 1 residues is supported by the AlphaFold2 (Jumper et al., 2021) predicted structure of EVA‐ACA1001 in complex with CCL7, in which the CC + 1 residue is tyrosine (Figure S6).

Finally, a shallow groove formed by the N‐loop and β3‐strand of CCL16 provides a binding site for the side chains of Phe23, Thr26, Pro28, and His31, which are part of an extended region at the junction between the N‐terminus and the core of EVA‐ACA1001 (Figure 6e). Among these evasin residues, both Thr26 and Pro28 are expected to be conformationally constrained due to their locations within the sequence Cys25‐Thr26‐Cys27‐Pro28. Moreover, these residues are conserved or substituted by other bulky hydrophobic residues in most class A3 evasins as well as many class A1 evasins (Bhusal et al., 2020). On the other hand, the interactions of EVA‐ACA1001 Phe23, sandwiched between CCL16 Tyr22 and Arg51 (Figure S7a,b), are absent in the chemokine‐bound structures of EVA‐AAM1001. These interactions may explain the small decrease in binding affinity for some chemokines upon removal of residues 1–24 from EVA‐ACA1001 (Figure 5).

## 3 DISCUSSION

This study reports the characterization of EVA‐ACA1001, which is only the second member of the class A3 evasins to be described. We found that EVA‐ACA1001 binds with high affinity to almost all CC chemokines. Moreover, the crystal structure of EVA‐ACA1001 in complex with CCL16 revealed features that are conserved among all class A evasins, others that are conserved only among class A3 evasins, and unique features of EVA‐ACA1001 that account for its broad‐spectrum chemokine binding.

Comparison of the EVA‐ACA1001:CCL16 complex to previously reported chemokine‐bound structures of the class A1 evasins EVA‐1 (Dias et al., 2009) and EVA‐P974 (Bhusal et al., 2022) and the class A3 evasin EVA‐AAM1001 (Devkota et al., 2023) reveals that the structural basis of CC motif recognition is conserved across all class A1 and A3 evasins. Specifically, in all cases, the chemokine CC motif forms at least four backbone‐backbone hydrogen bonds with the evasin β1 strand. Previously, we have shown that these four hydrogen bonds cannot form simultaneously if even a single amino acid is inserted between the cysteine residues of the CC motif (Bhusal et al., 2022). Thus, the current observations support our understanding that all class A evasins utilize a similar biomolecular mechanism to specifically recognize CC chemokines over other chemokine families (CXC, XC, and CX_3_C).

In contrast to the conserved role of the evasin β1 strand in CC motif recognition, the evasin N‐ and C‐termini play different roles in class A3 compared to class A1 evasins. In the class A3 evasins EVA‐ACA1001 and EVA‐AAM1001, the complete removal of the N‐ or C‐terminal residues had minimal effects on chemokine binding (Devkota et al., 2023). On the other hand, similar truncations of the class A1 evasin EVA‐P974 resulted in a loss of binding to most target chemokines (Bhusal et al., 2022). The importance of these regions in class A1 evasins is supported by studies of various chimeras (Aryal et al., 2022; Eaton et al., 2018), point mutants (Bhusal et al., 2022; Bonvin et al., 2014; Dias et al., 2009), and post‐translationally modified evasins (Franck et al., 2020). Thus, it appears that class A3 evasins have evolved from class A1 evasins such that they no longer utilize their N‐ and C‐terminal regions but can still bind with high affinity to numerous CC chemokines.

The ability of terminally‐truncated class A3, but not class A1, evasins to bind chemokines with high affinity most likely results from their well‐defined hydrophobic CC + 1 binding pocket. This pocket is absent (or less well‐defined) in class A1 evasins but is conserved and defined, in part, by the unique fifth disulfide bond, in class A3 evasins. Nevertheless, the CC + 1 pocket differs among the structurally characterized class A3 evasins, giving rise to differences in chemokine selectivity. In particular, whereas EVA‐AAM1001 is relatively selective for chemokines with aliphatic CC + 1 residues, EVA‐ACA1001 binds broadly to chemokines with both aliphatic and aromatic amino acids at the CC + 1 position. Structural comparison of these two evasins suggests that EVA‐ACA1001 residue Pro42, corresponding to EVA‐AAM1001 residue Leu39, may play an important role in opening the base of the pocket to form a tunnel through the protein structure (Figure 6d). Previous studies have suggested that such tunnels in proteins may exhibit relatively high structural flexibility (Stank et al., 2016). Thus, such flexibility may contribute to the broad‐spectrum chemokine binding of EVA‐ACA1001.

## 4 CONCLUDING REMARKS

We have characterized a new member of the class A3 evasin family, EVA‐ACA1001, as a high‐affinity, broad‐spectrum CC chemokine binder, and inhibitor. Whereas the structural basis of CC motif recognition is conserved across both class A1 and class A3 evasins, the evasin termini contribute substantially to chemokine recognition only in class A1 evasins. In contrast, the CC + 1 pocket is a defining characteristic of class A3 evasins and differs among these evasins, resulting in distinct spectra of chemokine targets. This structural understanding provides a rational basis to guide the engineering of evasins for a variety of diagnostic or therapeutic applications.

## 5 MATERIALS AND METHODS

### 5.1 Protein expression and purification

Proteins were expressed and purified according to the procedure reported by (Bhusal et al. 2022). Briefly, genes encoding EVA‐ACA1001 and CCL16 were purchased as gBlocks genes from Integrated DNA Technologies and cloned into a pET‐28a vector encoding a His_6_ tag followed by a Small Ubiquitin‐like Modifier (SUMO) tag at the protein N‐terminus. Plasmids were transformed into Rosetta gami2 (DE3) *E. coli* cells, and proteins were overexpressed by inducing 0.5 mM isopropyl β‐D‐1‐thiogalactopyranoside and growing for 18 h at 20°C. The soluble expressed EVA‐ACA1001 and CCL16 were purified in sequential steps: metal affinity chromatography, cleavage of the SUMO tag using ULP1 protease, metal affinity chromatography to remove the SUMO tag, size exclusion chromatography, and C4 reversed‐phase chromatography. The resulting proteins were lyophilized and stored at −80°C for further experiments.

### 5.2 Chemokine binding by SPR

For binding experiments, EVA‐ACA1001 and its variants were expressed with either an N‐terminal or C‐terminal linker (GGGGS)3 and an AVI tag (GLNDIFEAQKIEWHE) and purified as described above. Avi‐tagged EVA‐ACA1001 was reconstituted at 0.1–0.2 mg/mL in a buffer containing 10 mM Tris pH 8.0 and biotinylated using BirA ligase in the presence of 500 mM Bicine pH 8.3, 500 μM D‐biotin, 100 mM magnesium acetate, and 100 mM ATP and then purified by size exclusion chromatography. All SPR experiments were carried out at 25°C on a Biacore T100 instrument (Cytiva), using a Biotin CAPture kit (Cytiva) and running buffer 10 mM HEPES, 500 mM NaCl, 0.002% Tween 20, 3 mM EDTA, 1 mg/mL carboxymethyl dextran, pH 7.5. The biotinylated EVA‐ACA1001 was immobilized on a streptavidin‐coated CAPture chip by flowing 0.2 μM evasin for 60 s at a flow rate of 10 μL/min to obtain a signal of 80 response units. All available chemokines were screened, using single‐cycle kinetics, for binding to the immobilized evasin, by flowing 500 nM of chemokine for 240 s at 30 μL/min, followed by a dissociation time of 600 s. For chemokines that exhibited binding in the screen, the binding affinities were determined by sequentially injecting five two‐fold increasing concentrations of chemokines (31.25–500 nM) for 240 s at 30 μL/min, all followed by a dissociation time of 600 s. The data were fit to a 1:1 binding model in Biacore T100 evaluation software. Experiments were performed three times independently.

### 5.3 Crystallization and structure determination

Lyophilized EVA‐ACA1001 and CCL16 were reconstituted in 10 mM HEPES pH 7.5, 150 mM NaCl, and 5% glycerol and mixed in a 1:1.2 ratio. The complex was purified using a Superdex 75‐size exclusion chromatography column (Cytiva) in a buffer of 10 mM HEPES pH 7.5, 150 mM NaCl, and concentrated to ~20 mg/mL and subjected to initial crystallization trials in commercial screens (JCSG‐plus and Wizard; Molecular Dimensions; Index and Crystal 20; Hampton Research) at the Monash Macromolecular Crystallization Facility. Initial hits were obtained at 0.1 M HEPES pH 7.0, 0.5% v/v Jeff ED‐2001, 1.1 M sodium malonate, and optimized to diffracting quality crystals using the microbatch crystallization method with 50:50 paraffin: silicon oil (Hampton Research). Diffraction data were collected using the MX2 beamline at the Australian Synchrotron. All data sets were indexed and processed using XDS (Kabsch, 2010) and scaled with AIMLESS using the CCP4 program suite (Winn et al., 2011). The EVA‐ACA1001:CCL16 structure was solved by molecular replacement using MRage (Phenix Suite) (Adams et al., 2010) with EVA‐AAM1001:CCL16 (PDB: 7SCT) as a search model. The initial model building was subjected to iterative auto‐building cycles using BUCCANEER (Cowtan, 2006). Subsequently, the model at 2.6 Å was completed by several rounds of refinement using phenix.refine (Adams et al., 2010) and manual building using COOT (Emsley & Cowtan, 2004). The stereochemical quality of the model was analyzed using MolProbity (Williams et al., 2018). The full data collection and refinement statistics are in Table 3. The final coordinate and structure factor files are deposited into the Protein Data Bank with accession code 8FK9.

### 5.4 cAMP inhibition assay

Functional inhibition of chemokines by EVA‐ACA1001 was carried out using the cAMP bioluminescence resonance energy transfer (BRET) biosensor (CAMYEL) assay (Jiang et al., 2007). Flp‐In‐CHO cells stably expressing CCR2 or CCR5 were transiently transfected with the CAMYEL biosensor (Lim et al., 2021) and grown overnight in Dulbecco's Modified Eagle Medium supplemented with 5% fetal bovine serum and 1% penicillin/streptomycin at 37°C and 5% CO_2_. The cells were transferred to a 96‐well plate (50,000 cells/well) and incubated at 37°C 5% CO_2_ overnight. The next day, cells were washed twice and equilibrated with Hank's Balanced Salt Solution for 30 min at 37°C, then incubated for 10 min with Rluc substrate coelenterazine‐h (Nanolight Technology) at a final concentration of 5 μM. For concentration response curves (to determine the chemokine *EC*_*80*_), cells were treated with different concentrations of chemokines (CCL7 for CCR2; CCL3, CCL4, or CCL8 for CCR5) for 10 min, followed by the addition of forskolin (Sigma‐Aldrich) at a final concentration of 10 μM for 10 min to induce cAMP production. For EVA‐ACA1001‐mediated inhibition, the cells were treated with chemokines (concentration approximately *EC*_*80*_), that is, 100 nM CCL2; 60 nM CCL3; 80 nM CCL4; or 100 nM CCL8, alone or pre‐incubated with the different concentrations of EVA‐ACA1001 for 10 min, followed by forskolin (final concentration 10 μM) for 10 min. The Rluc and yellow fluorescent protein emissions were then measured at 475 and 535 nM, respectively, using a BRET one‐plus module in a BMG Labtech PHERAstar FS plate reader, and the BRET ratio was calculated. The change in BRET ratio is expressed as the percentage inhibition of forskolin‐induced cAMP production by chemokines. The data presented are the mean ± SEM of at least three independent measurements.

## AUTHOR CONTRIBUTIONS

**Shankar Raj Devkota:** Conceptualization; writing – original draft; writing – review and editing; methodology; formal analysis; validation; investigation. **Pramod Aryal:** Methodology; writing – review and editing; formal analysis; investigation; conceptualization. **Matthew C. J. Wilce:** Conceptualization; writing – review and editing; formal analysis; validation. **Richard J. Payne:** Conceptualization; writing – review and editing; funding acquisition. **Martin J. Stone:** Conceptualization; supervision; project administration; writing – review and editing; writing – original draft; funding acquisition; formal analysis; methodology. **Ram Prasad Bhusal:** Conceptualization; funding acquisition; writing – original draft; writing – review and editing; supervision; project administration; formal analysis; methodology.

## CONFLICT OF INTEREST STATEMENT

The authors declare no competing interests.

## Supporting information


**Data S1.** Supporting information.

## Data Availability

The coordinates and structure factors have been deposited in the Protein Data Bank under the following accession code: 8FK9.
