# Supplementary material for: Structural basis of chemokine recognition by the class A3 tick evasin EVA‐ACA1001
Source: Protein Sci. 2024 May 9;33(6):e4999. doi: 10.1002/pro.4999 (PMC11081419; doi:10.1002/pro.4999)
Supplement: Supplementary file 1 — Data S1. Supporting information. [file PRO-33-e4999-s001.docx]

# **Supplementary material**

**Structural basis of chemokine recognition by the class A3 tick evasin EVA-ACA1001**

Shankar Raj Devkota^1^, Pramod Aryal^1^, Matthew C.J. Wilce^1^, Richard J. Payne^2,3^, Martin J. Stone^1,*^ and Ram Prasad Bhusal^1,*^

^1^Monash Biomedicine Discovery Institute, and Department of Biochemistry and Molecular Biology, Monash University, Clayton, VIC 3800, Australia

^2^ School of Chemistry, The University of Sydney, Sydney, NSW 2006, Australia

^3^ Australian Research Council Centre of Excellence for Innovations in Peptide and Protein Science, The University of Sydney, Sydney, NSW 2006, Australia

*Correspondence: martin.stone@monash.edu; Tel.: +61-3-9902-9246; ram.bhusal@monash.edu; Tel.: +61-3-9905-5337


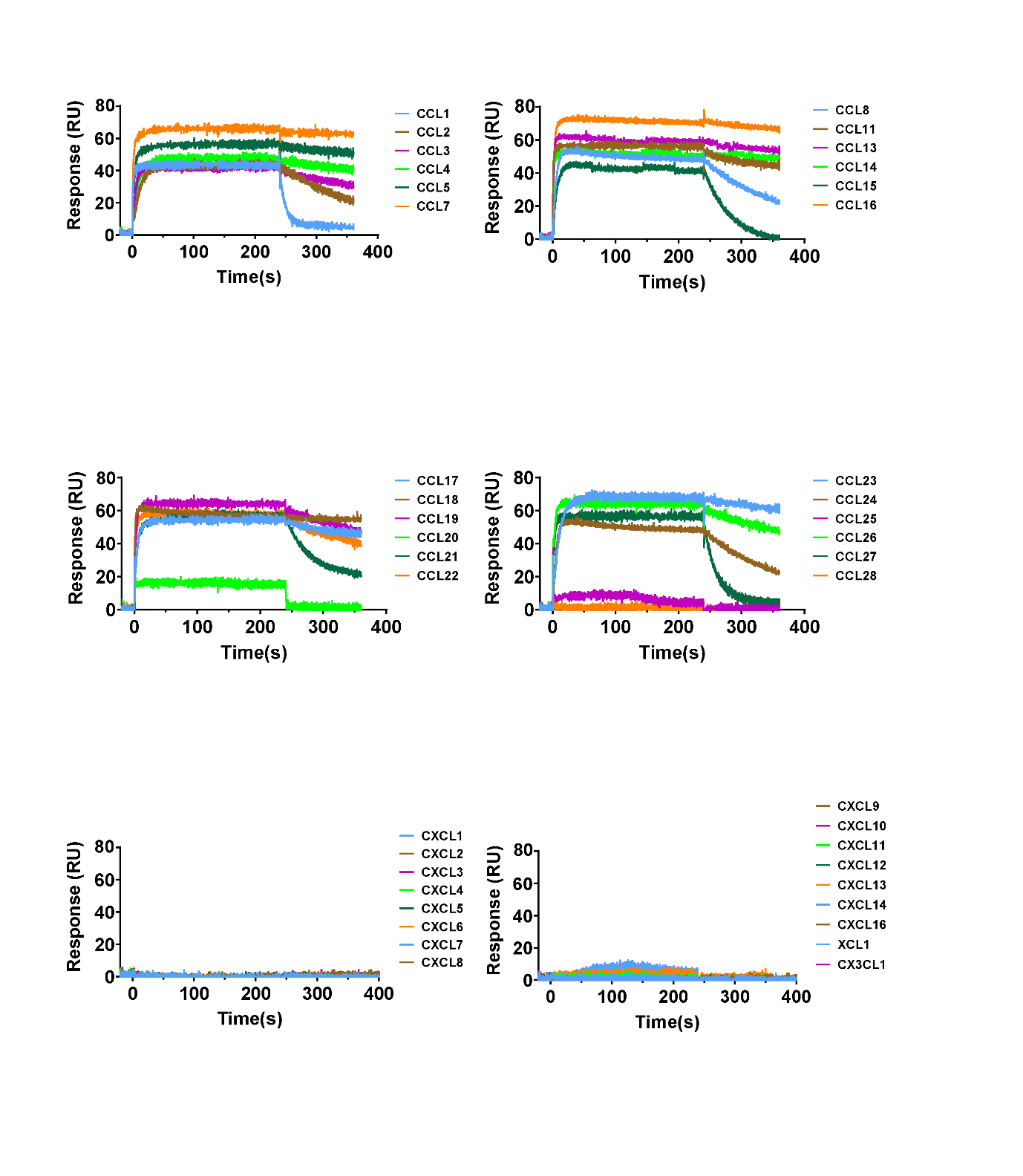


***Figure S1. EVA-ACA1001*** ***selectively binds to multiple human CC chemokines.*** Screening of EVA-ACA1001 with multiple human chemokines by SPR using single-cycle kinetics. Sensorgrams show the binding of immobilized EVA-ACA1001 to a single chemokine concentration (500 nM). EVA-ACA1001 selectively binds to CC chemokines but not to other classes of chemokines.


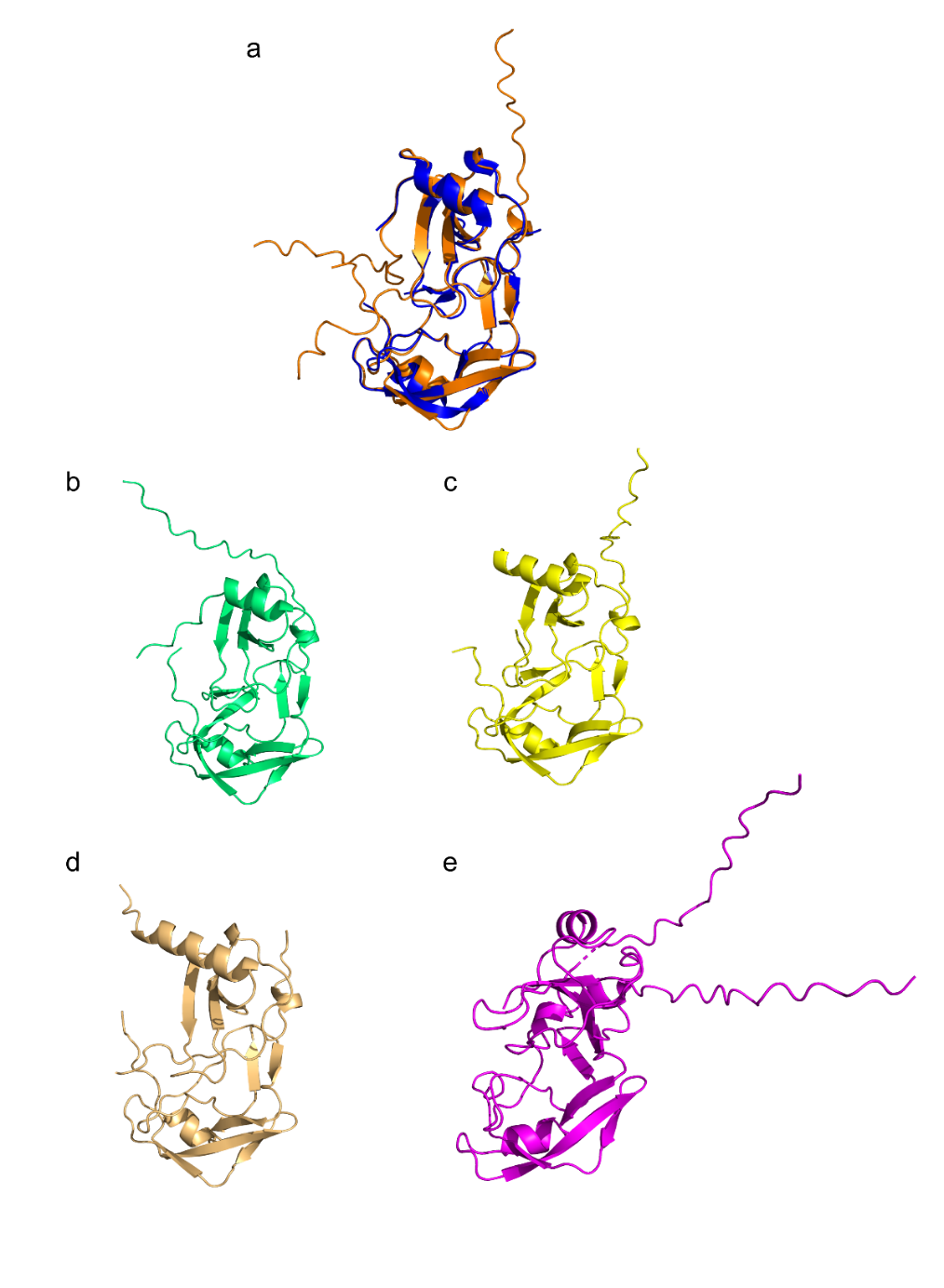


**Figure S2. AlphaFold2 predicted structure of EVA-ACA1001 with chemokines.** (a) Structural alignment of EVA-ACA1001 CCL16 (blue) structure and AlphaFold2 predicted structure of EVA-ACA1001 CCL16 (TV orange) shows excellent agreement with root-mean-square deviation (rmsd) of 0.582 Å for 133 C_α_ atoms. (b-e) AlphaFold2 predicted strucutre of EVA-ACA1001 with differnet human chemokines shows the same fold like experimental strucuture of EVA-ACA1001 CCL16. (b) EVA-ACA1001 CCL1. (c) EVA-ACA1001 CCL5 (d) EVA-ACA1001 CCL7 (e) EVA-ACA1001 CCL15.

***
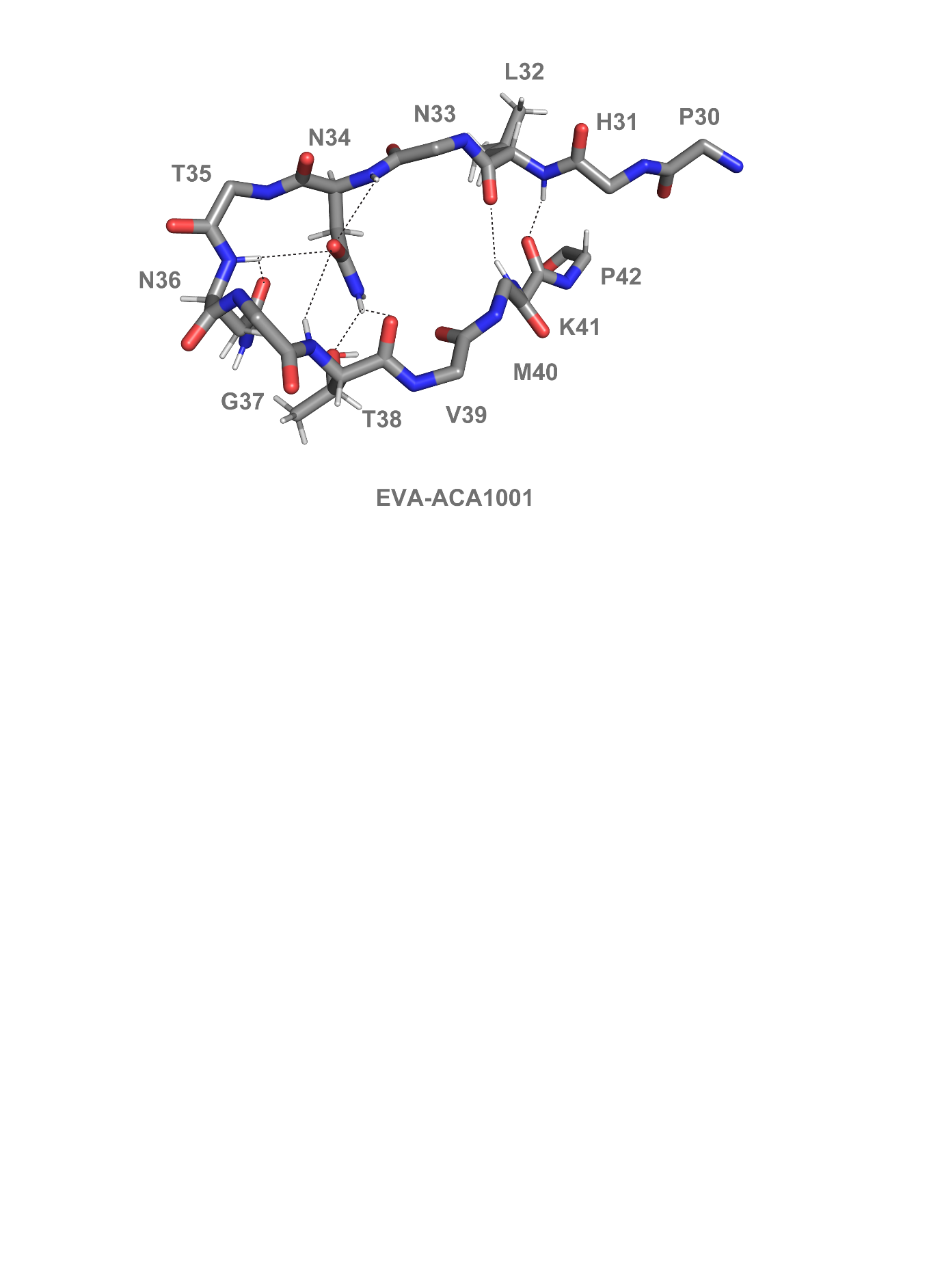
***

***Figure S3. EVA-ACA1001 hairpin-like structure at equivalent positions of β1 and β2 strands of other evasin structures.*** Possible hydrogen bond interactions (both between backbones and backbones to sidechains) in this specific region are shown with dashed lines.


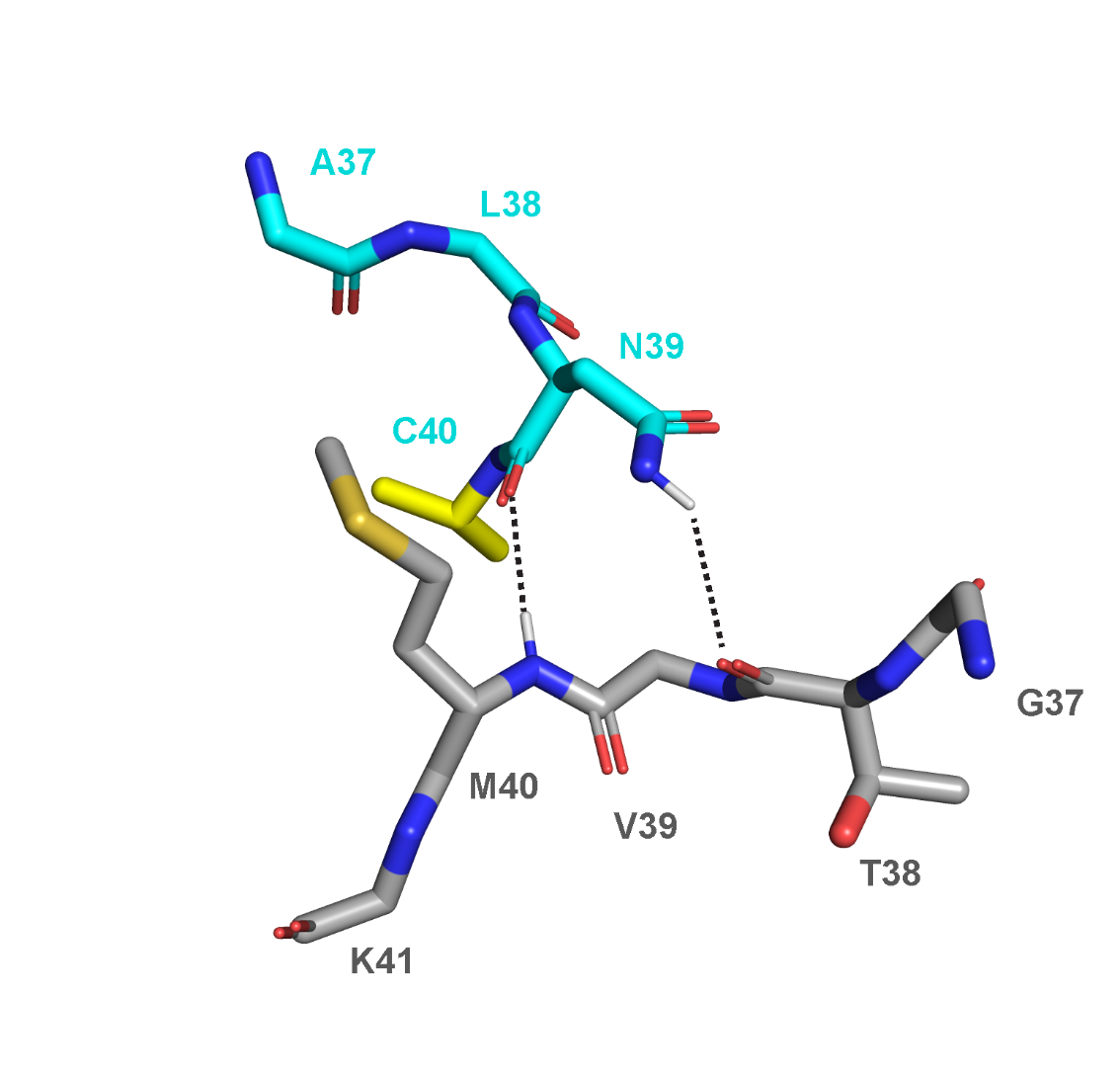


**CCL16**

**EVA-ACA1001**

***Figure S4. Additional hydrogen bonds between EVA-ACA1001 and CCL16.*** Shown are a backbone-backbone hydrogen bond between EVA-ACA1001 Met40 and CCL16 Asn39 and a backbone to side chain hydrogen bond between EVA-ACA1001 Thr38 and CCL16 Asn39.


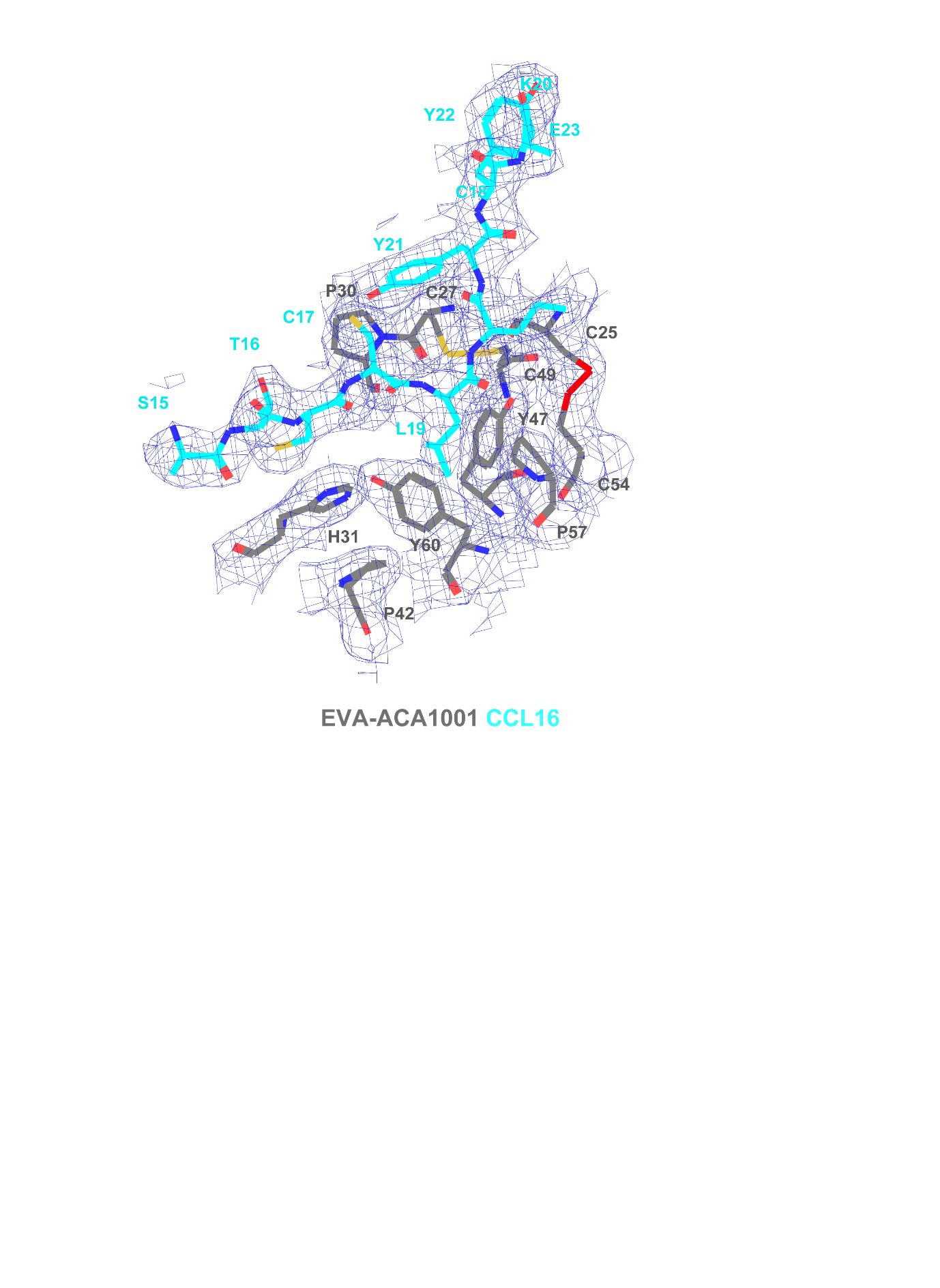
­­

***Figure S5. 2Fo-Fc composite omit map showing electron density of the EVA-ACA1001 hydrophobic pocket interacting to CCL16 CC+1 residue.*** The composite omit 2Fo-Fc maps were contoured at 1σ on Phenix and visualised using PyMOL as a blue mesh. Amino acid residues of EVA-ACA1001 hydrophobic pocket (gray) and CCL16 S15-E23, including the CC+1 residue Leu19 (cyan) are shown as sticks.

**
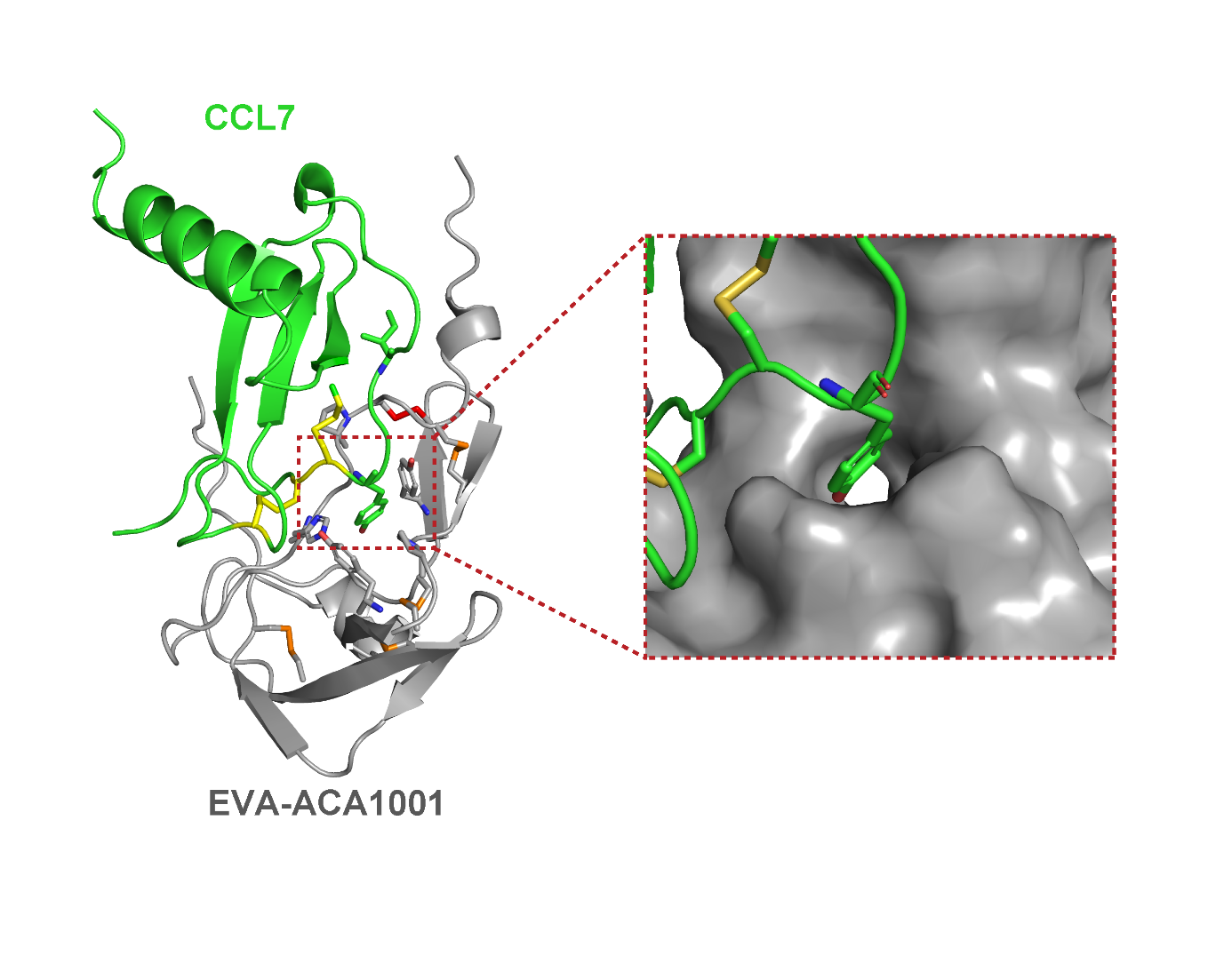
*Figure S6. Predicted structure of EVA-ACA1001:CCL7.*** AlphaFold2-predicted structure of EVA-ACA1001 (grey) in complex with CCL7 (green). Inset: EVA-ACA1001 (surface) and CCL16 (cartoon and stick) demonstrate the proper fitting of the CC+1 residue of CCL7 (Tyr13) into the hydrophobic pocket of EVA-ACA1001.


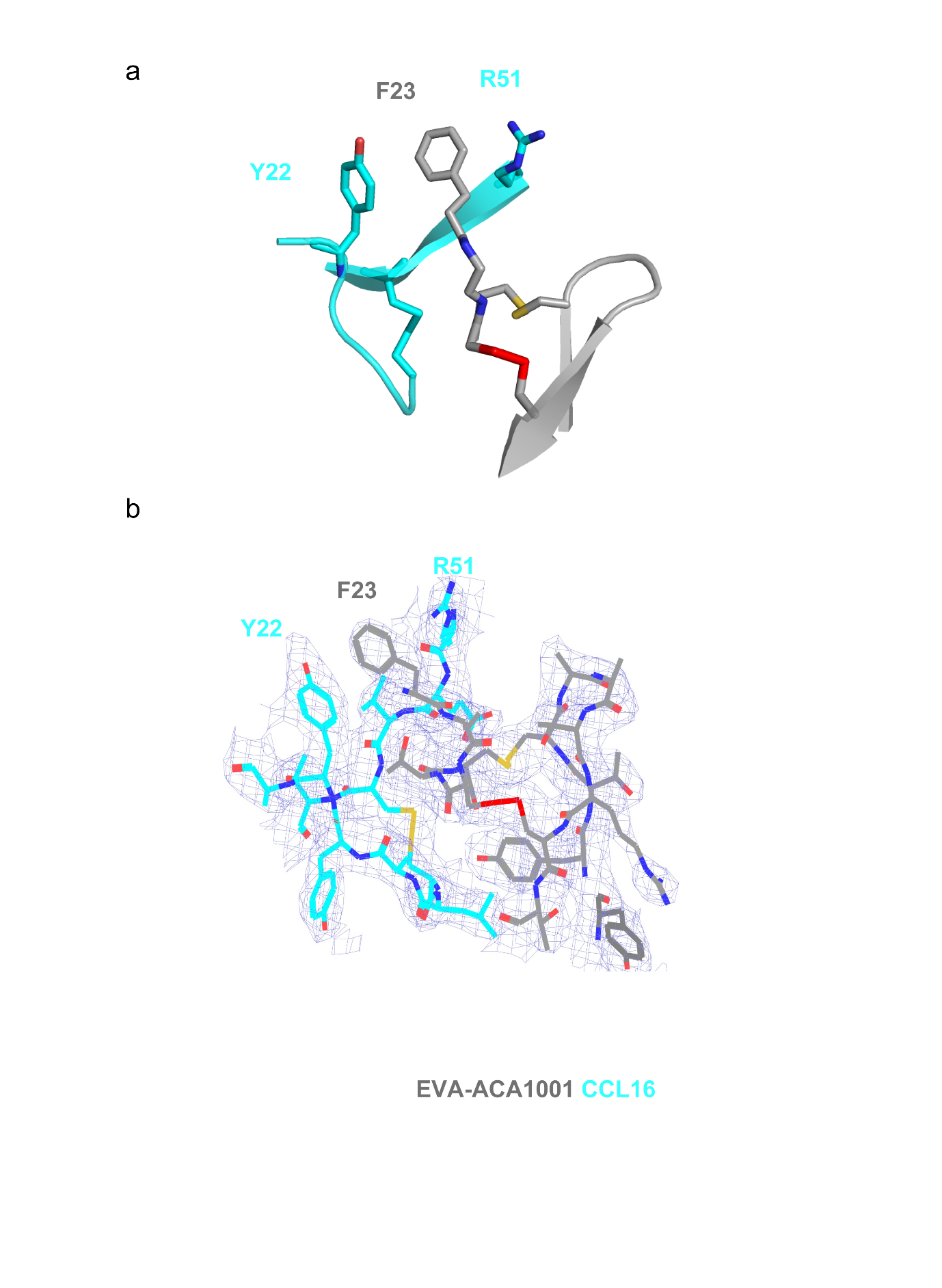


***Figure S7. Interactions of EVA-ACA1001 Phe23 with CCL16****.* (a) Phe23, near the end of the N-terminal region of EVA-ACA1001 (gray) is sandwiched between Arg51 and Tyr22 of CCL16 (cyan). The first two disulfides bonds of EVA-ACA1001 (red and yellow) help to define the orientation of Phe23. (b) 2F_o_-F_c_ composite omit electron density map of panel a. The composite omit 2Fo-Fc maps were contoured at 1σ on Phenix and visualised using PyMOL as a blue mesh. Amino acid residues of EVA-ACA1001 (gray) and CCL16 (cyan) are shown as sticks.
